# Abnormal nucleoli architecture and aggregate formation in nucleophosmin mutated acute myeloid leukaemia

**DOI:** 10.1242/jcs.263553

**Published:** 2025-05-21

**Authors:** Martin Grundy, Kellie Lucken, Xiaomeng Xing, Eva L. Simpson, Alice Worker, Ahmed Bayyoomi, Alison J. Beckett, Ian A. Prior, Daniel G. Booth, Claire H. Seedhouse

**Affiliations:** ^1^ Translational Medical Sciences, Biodiscovery Institute, University of Nottingham, Nottingham NG7 2RD, United Kingdom; ^2^ Molecular Physiology and Cell Signalling, Institute of Systems, Molecular and Integrative Biology, University of Liverpool, Liverpool L69 7BE, UK

**Keywords:** Acute myeloid leukaemia, Nucleophosmin, Mutations, Nucleoli, Aggregates

## Abstract

Mutations in the nucleophosmin (*NPM1*) gene represent the most common genetic alteration in acute myeloid leukaemia (AML) and result in mis-localisation of the mutated protein from a predominantly nucleolar localisation to a predominantly cytoplasmic distribution. Here, we use high resolution imaging to demonstrate that NPM1 is crucial for maintaining normal nucleoli architecture and specifically the integrity of the enigmatic nucleoli rim, the least understood nucleolar compartment. We demonstrate that cell lines and primary cells with NPM1 mutations from individuals with AML have aberrant nucleoli architecture; intriguingly this abnormal nucleolar phenotype is reversible. Using a surrogate for rRNA synthesis, we show that the aberrant phenotype is associated with differences in nucleolar function; specifically, activity of RNA polymerase I is increased in NPM1 mutated cells. Perinucleolar chromatin organisation is also markedly different in NPM1 mutant cells. Finally, we report the novel finding that NPM1 mutated protein forms distinct aggregates and characterise these for the first time. This work reveals how nucleolar organisation contributes to the molecular mechanisms underpinning NPM1-driven AML, revealing novel therapeutic vulnerabilities.

## Teaser

**Highlighted Article:** Nucleophosmin (NPM1) mutated acute myeloid leukaemia cells have aberrant nucleoli architecture and function along with perinucleolar heterochromatin reorganisation. NPM1 mutated protein also forms distinct aggregates.

## INTRODUCTION

Acute myeloid leukaemia (AML) is characterised by the clonal proliferation of undifferentiated myeloid blast cells, which can infiltrate the bone marrow, blood and other tissues leading to ineffective haematopoiesis, bone marrow failure and severe cytopenia. Although people of all ages can develop AML, it is generally a disease of the elderly with a median age at diagnosis of 68 years (DiNardo et al., 2023). Despite advances in supportive care and relapse monitoring, prognosis remains dismal, with only 9.4% of patients who are 65 years and older at diagnosis achieving a 5-year survival (DiNardo et al., 2023; Dohner et al., 2015). Unlike many cancers, the prognosis for AML has shown little improvement in over 20 years. There is clearly an unmet need to better understand the molecular mechanisms that trigger and drive leukaemia in these individuals, providing a tractable approach for discovery of new therapeutic targets. Mutations in the nucleophosmin (*NPM1*) gene represent the most common genetic alteration in AML, occurring in ∼35% of adult AMLs (Falini et al., 2005). NPM1 is a ubiquitously expressed and functionally diverse phosphoprotein with reported roles in ribosome biogenesis and transport, DNA repair, centrosome duplication and as a protein chaperone (Murano et al., 2008; Okuda, 2002; Yu et al., 2006). Correct subcellular localisation of NPM1 is crucial for normal cellular homeostasis. NPM1 routinely shuttles between the cytoplasm and nucleus (Borer et al., 1989), but under normal physiological conditions NPM1 localises predominantly to the nucleolus (Nishimura et al., 2002), the largest sub-compartment of the nucleus.

First observed more than 200 years ago, nucleoli are membrane-less subnuclear compartments that are the cellular site for ribosome synthesis and assembly and additionally comprise proteins involved in stress response and cell cycle regulation (Boisvert et al., 2007). The lack of enclosing membrane allows nucleoli to respond dynamically to cellular signals by changing their size and protein composition. Nucleoli form around pre-ribosome RNA gene repeats, termed nucleolar organiser regions (NORs). In humans, NORs are located on the short arms of acrocentric chromosomes 13, 14, 15, 21 and 22 (Pederson, 2011). The nucleolus, which is formed by liquid–liquid phase separation, has long been considered as having three droplet-like layers of varying miscibility: the fibrillar centre (FC), the dense fibrillar component (DFC) and the granular component (GC). More recently, comprehensive spatiotemporal mapping of nucleolar architecture has revealed a fourth nucleoli sub-compartment, the nucleoli rim, and it has been reported that 157 nucleoli proteins, including NPM1, have a characteristic nucleoli rim-like localisation (Stenstrom et al., 2020).

Mutations in the *NPM1* gene represent the most common (>30% of individuals) genetic alteration in AML. *NPM1* insertion mutations are AML specific and almost always occur at exon 12, causing a frameshift in the C-terminus encoding region, which hampers the correct folding of NPM1 (Liso et al., 2008). The altered reading frame leads to loss of the nucleolar localisation signal (NLS) and generation of an additional nuclear export signal (NES), thought to increase binding to the nuclear export protein XPO1, ultimately resulting in the aberrant cytoplasmic distribution of the mutated protein (Bolli et al., 2007; Falini et al., 2009, 2006). More than 50 different mutations in the *NPM1* gene have been identified, all of which result in mis-localisation of the mutated protein (denoted NPM1c+) to the cytoplasm (Heath et al., 2017). This cytoplasmic mis-localisation of NPM1c+ also leads to HOX gene upregulation which is crucial for leukemogenesis and is required for disease maintenance (Brunetti et al., 2018). Most studies of NPM1 mutated AML have focussed on the aberrant localisation of the mutated protein from the nucleolus to the cytoplasm, along with factors that are expelled from the nucleus to the cytoplasm in consort, including CTCF, FBW7γ and PU.1 (also known as SPI1) (Bonetti et al., 2008; Gu et al., 2018; Wang et al., 2020). This includes efforts to reverse NPM1c+ mis-localisation with selective inhibitor of nuclear export (SINE) compounds, such as Selinexor and Eltanexor, but these have so far resulted in limited clinical benefit (Garzon et al., 2017; Pianigiani et al., 2022). More recently, attention has shifted towards the nucleus, with studies showing that mutant NPM1 binds to specific chromatin regions, where it directly regulates oncogenic gene expression (Uckelmann et al., 2023; Wang et al., 2023). Owing to stalled progress in development of therapeutic targets for AML and reflecting on new advances mapping nucleolar architecture, we identified a clear need to revisit the molecular dynamics of NPM1 in the context of AML. Using high-resolution imaging we performed a comprehensive survey of wild-type (wt) and mutant NPM1 subcellular distribution, revealing multiple novel features, including the role of NPM1wt in maintaining normal nucleoli architecture, and demonstrate the loss of nucleoli rim structure and distorted nucleoli following NPM1wt knockdown. We report for the first time that this aberrant nucleolus phenotype is also present in NPM1 mutated cell lines and primary samples and demonstrate that the phenotype is reversible. Nucleoli from NPM1 mutant cells also have functional differences and changes in chromatin organisation. Finally, we also report the intriguing finding that NPM1 mutated protein forms distinct aggregates in NPM1 mutated cells and perform the first characterisation of these enigmatic structures. This work not only contributes to our understanding of the molecular mechanisms underpinning NPM1-driven AML but also reveals an unexpected novel vulnerability to be exploited for therapeutic intervention.

## RESULTS

### NPM1 wild-type knockdown results in loss of the nucleoli rim and nucleoli distortion

**Fig. 1.**
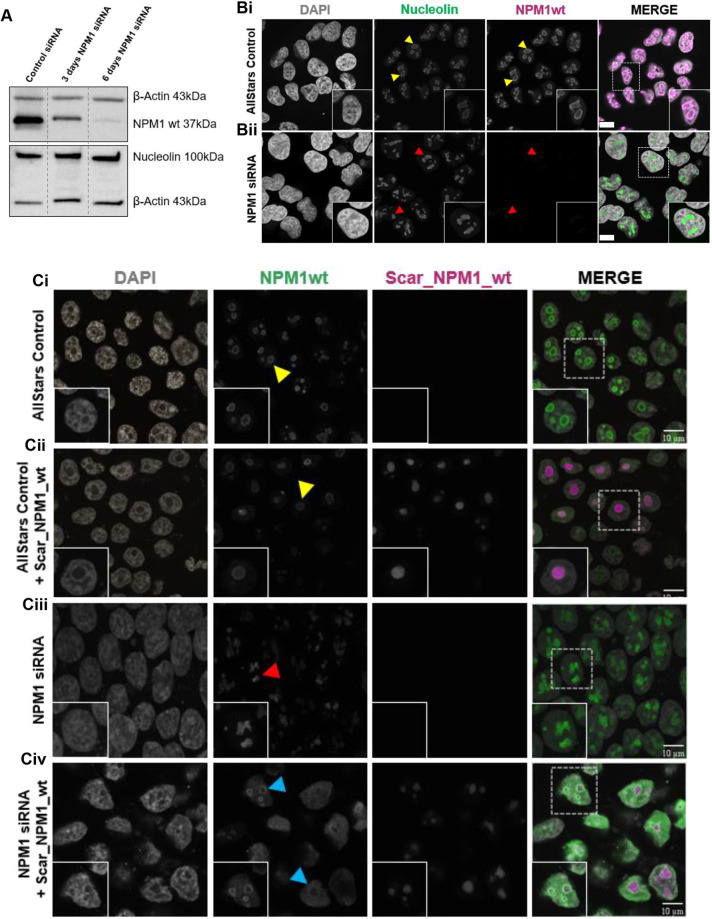
**NPM1 wild-type knockdown results in loss of the nucleoli rim and nucleoli distortion.** (A) NPM1wt and nucleolin protein expression in Hela cells following 3 or 6 days NPM1wt siRNA interference. (B) Hela cells were stained with DAPI and antibodies against NPM1wt and nucleolin following 6 days NPM1wt siRNA interference or AllStars negative control. Normal NPM1wt and nucleolin nucleoli rim staining (yellow arrowheads). Cells lacking a nucleoli rim with distorted nucleoli (red arrowheads). Scale bars: 10 µm. (C) HEK-293T cells were subjected to 2 days NPM1wt siRNA interference followed by 24 h transfection with Scar_NPM1_wt and then stained with DAPI and NPM1wt antibody. Normal NPM1wt nucleoli rim localisation (yellow arrowheads) and distorted nucleoli following NPM1wt siRNA interference (red arrowheads). Cells with normal NPM1wt nucleoli rim localisation following Scar_NPM1_wt ectopic rescue (blue arrowheads). All imaging was performed using a Leica DMI4000 B confocal microscope. Images representative of three experimental repeats.

Previously, some studies have reported that NPM1 depletion can cause perinucleolar heterochromatin rearrangement (and the disruption of epigenetic marks) (Holmberg Olausson et al., 2014), and others have shown that NPM1 depletion results in loss of general nucleolar structure (Holmberg Olausson et al., 2014; Okuwaki et al., 2021). However, confirmation of NPM1 distribution, function and consequences of manipulation (via depletion or expression of mutants), explicitly relating to the nucleolar rim, remains elusive. To address this, we conducted targeted NPM1 depletion using siRNA followed by microscopy analysis. Western blotting and band densitometry (data not shown, *n*=3) revealed a 99% reduction in NPM1 abundance following a 6-day RNAi period (Fig. 1A). Importantly, expression levels of nucleolin, another nucleoli rim localising protein (Stenstrom et al., 2020), remained unchanged, underscoring the specificity of NPM1 knockdown (Fig. 1A). Immunofluorescence imaging confirmed clear NPM1 and nucleolin localisation at the nucleoli rim (Fig. 1Bi, yellow arrowheads). Neither protein was present at the nucleolar rim following 6 days of NPM1 RNAi. Instead, knockdown of NPM1 resulted in distorted nucleoli and a diffuse staining pattern of both nucleolin and the residual NPM1 within the remaining nucleolar space (Fig. 1Bii, red arrowheads). This suggests that NPM1 has a pivotal role in maintaining nucleoli rim structure. Next, we performed rescue experiments to test whether loss of nucleolar integrity was reversible. Following 2 days of NPM1 RNAi, cells were transfected to express an mScarlet-tagged version of NPM1wt (Scar_NPM1_wt). Remarkably, re-expression of NPM1wt resulted in almost complete correction of normal nucleolar phenotype (compare Fig. 1Civ, blue arrowheads, with Fig. 1Ci, yellow arrowhead), confirming the essential role of NPM1 in maintaining normal nucleoli rim structure. Interestingly, experimental controls revealed that ectopic overexpression of NPM1 (on a control RNAi background), regularly caused the formation of large single nucleoli, but that even in this instance NPM1wt was still mostly detectable at the nucleolar rim (Fig. 1Cii, yellow arrowhead). This work supports previous studies and suggests that there is likely a stringent cellular tolerance of NPM1 and that normal NPM1 function is tightly coupled to appropriate abundance.

### Subcellular localisation of wild-type and mutant NPM1 revealed by high-resolution confocal microscopy

**Fig. 2.**
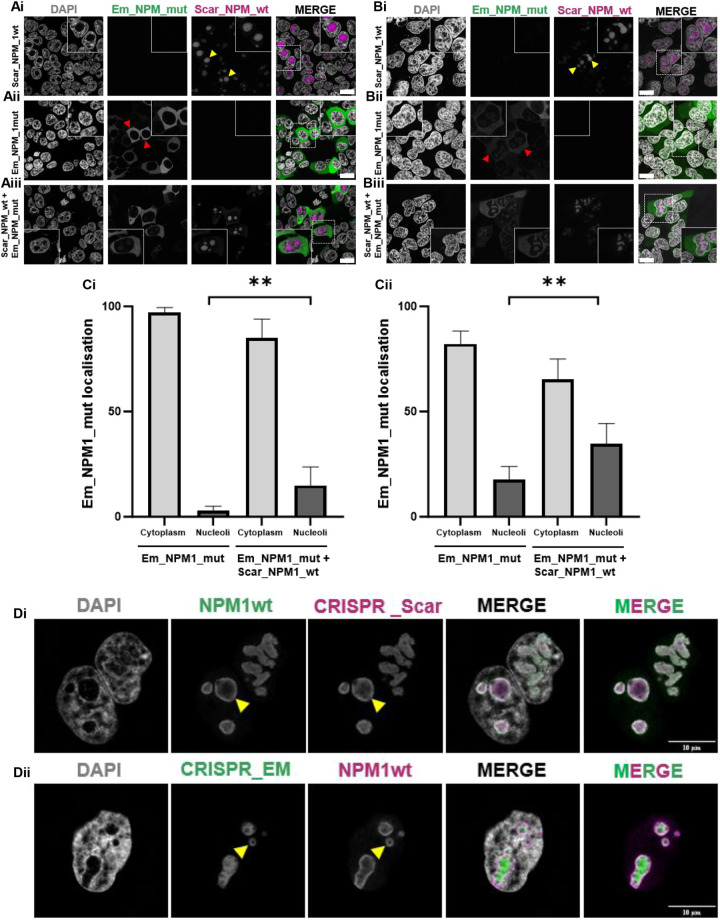
**Subcellular localisation of wild-type and mutant NPM1 revealed by high-resolution confocal microscopy.** (A) HEK-293T and (B) HeLa cells were transfected with Scar­_NPM1_wt and Em_NPM1_mut constructs for 24 h. Yellow arrowheads indicate NPM1wt localisation in the nucleoli. Red arrowheads indicate NPM1 mutant localisation to the cytoplasm. Scale bars: 10 µm. (C) Subcellular localisation of Em_NPM1_mut in HEK-293T (i) and HeLa cells (ii) was quantified using Fiji. Columns, mean data from 20 representative cells: bars, s.d. **P≤0.005 (unpaired two-tailed *t*-test). (D) mScarlet (Di) and mEmerald (Dii) fluorescently tagged NPM1wt knock-in HeLa cells were created by CRISPR-Cas9 gene editing. Cells were assessed using either the fluorescent tag or stained using antibodies against NPM1wt. Yellow arrowheads indicate NPM1wt localisation to the nucleoli rim. Images in D representative of three experimental repeats.

Next, to determine the impact of NPM1 mutation on nucleolar architecture, we closely examined the subcellular localisation patterns of both NPM1wt and the NPM1c+ mutant, using high resolution confocal microscopy. Initially, we chose a simplified system expressing mScarlet-tagged NPM1wt (Scar_NPM1_wt) or mEmerald-tagged NPM1c+ mutant (Em_NPM1_mut) in both HEK-293T cells (Fig. 2A) and HeLa cells (Fig. 2B). As expected, NPM1wt localised to the nucleoli (yellow arrowheads) whereas NPM1c+ predominantly localised to the cytoplasm (red arrowheads) in both cell types (Fig. 2A,B). Interestingly, a subtle difference was observed in the nucleolar-to-cytoplasmic distribution of NPM1c+, between the two cell types. In HEK-293T cells NPM1c+ was almost entirely cytoplasmic, whereas in HeLa cells a significant fraction (17.8%) localised to the nucleolus (Fig. 2C). This cell type variability is consistent with previous reports, suggesting that the discrepancy might be due to higher endogenous NPM1 expression in HeLa cells compared with HEK-293T cells (Brodska et al., 2017). Co-transfection (Scar_NPM1_wt+Em_NPM1_mut) resulted in an increased presence of NPM1 mutant protein in the nucleoli of both HEK-293T (435.3% increase, *P*=0.0043) and HeLa (95.2% increase, *P*=0.002) cells compared to single transfection (Em_NPM1_mut only). This supports the tug-of-war hypothesis, which proposes that the ratio of both wild-type and mutant constructs affect their localisation due to the formation of heterooligomers (Bolli et al., 2009). Curiously, we did not observe the characteristic nucleoli rim localisation of ectopic NPM1wt (Fig. 2A,B) that was readily apparent with antibody staining (Fig. 1B). We hypothesise that this discrepancy could either be a result of overexpression or due to fluorescent reporter (mScarlet tag) interference. To test this, we used CRISPR-Cas9 methodology to edit the *NPM1* gene, inserting a mScarlet or mEmerald fluorescent protein, at the endogenous locus. Fluorescence imaging of the engineered cell lines, co-labelled with anti-NPM1wt antibody, confirmed appropriate nucleoli rim localisation (Fig. 2D, yellow arrowheads). This serves as an important technical observation for the wider NPM1 field, demonstrating that NPM1 protein can tolerate reporter fusions, but only when expressed at endogenous, or near-endogenous levels. To confirm that the nucleoli rim staining is not an antibody, fixation or permeabilisation artifact, we next performed live imaging microscopy of HeLa or HEK-293T cells transiently expressing Scar_NPM1_wt. Live imaging revealed a slightly less distinct, but still clearly distinguishable, NPM1 rim localisation (Fig. S1) consistent with a previous report studying Ki-67 at the nucleolar rim (Stenstrom et al., 2020). Importantly, we were able to demonstrate that rim localisation was maintained over a period of 6 h (Movie 1). We also include the caveat that clear rim visualisation is dependent on the *z*-axis of the image as demonstrated in Fig. S1C.

### NPM1-mutated cells lack a nucleoli rim and have distorted nucleoli compared to NPM1 wild-type cells

We next investigated the subcellular localisation and impact of NPM1wt and NPM1c+ on nucleolar architecture in leukaemia cell lines. We used OCI-AML3 and IMS-M2 cell lines, both of which are heterozygous for NPM1wt and NPM1c+. OCI-AML2 cells, expressing only NPM1wt, were used as a reference control.

**Fig. 3.**
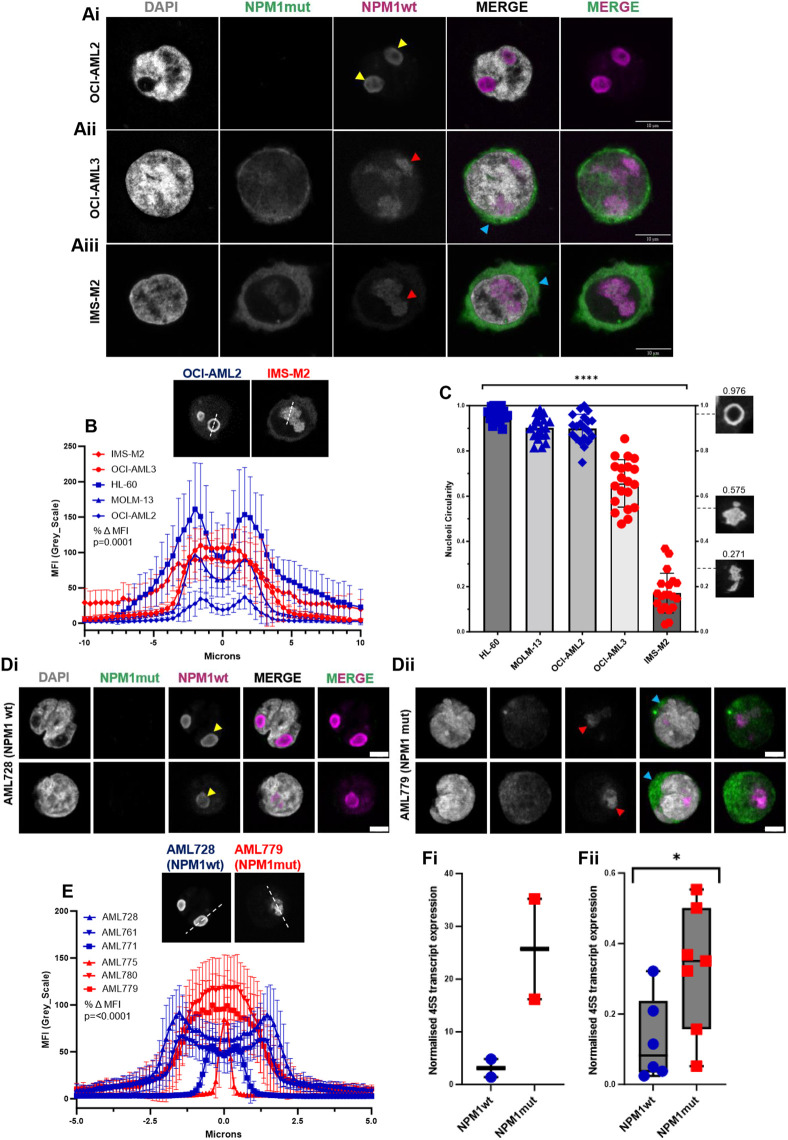
**NPM1 mutated cells lack a nucleoli rim and have distorted nucleoli compared to NPM1 wild-type cells.** (A) AML cell lines were stained with DAPI and antibodies against NPM1 mutant and NPM1wt. Yellow arrowheads indicate NPM1wt localisation to the nucleoli rim in NPM1 wild-type OCI-AML2 cells (Ai). Red arrowheads indicate a lack of nucleoli rim and distorted nucleoli in the OCI-AML3 (Aii) and IMS-M2 (Aiii) NPM1 mutated cells. Blue arrowheads indicate NPM1 mutant localisation to the cytoplasm. (B) NPM1wt localisation in the nucleolus of AML cell lines was quantified using the line scan tool in Fiji. NPM1 wild-type cell lines in blue, NPM1 mutant cell lines in red, mean of 20 nucleoli for each cell line: bars, s.d., %ΔMFI (high/low; MFI, mean fluorescence intensity) for NPM1 mutant lines compared to wild-type was *P*=0.0001 (unpaired two-tailed *t*-test). (C) AML cell line nucleoli circularity was measured using the threshold tool in Fiji. Columns, mean of 20 nucleoli for each cell line: bars, s.d. ****P≤0.0001 (NPM1 wild-type compared to mutant AML cell line circularity; unpaired two-tailed *t*-test). (D) Primary AML samples were stained with DAPI and antibodies against NPM1 mutant and NPM1wt. Yellow arrowheads indicate NPM1wt localisation to the nucleoli rim in NPM1 wild-type samples (Di). Red arrowheads indicate a lack of nucleoli rim and distorted nucleoli in the NPM1 mutated samples (Dii). Blue arrowheads indicate NPM1 mutant localisation to the cytoplasm. Scale bars: 10 µm. (E) NPM1wt localisation in the nucleolus of primary samples. NPM1 wild-type samples in blue, NPM1 mutant samples in red. Columns mean of 20 nucleoli for each sample: bars, s.d., %ΔMFI (high/low) for NPM1 mutant samples compared to wild-type was *P*=0.0001 (unpaired two-tailed *t*-test). (Fi) AML cell line or (Fii) primary sample 45S transcript expression, quantified using RT-PCR. The box represents the 25-75th percentiles, and the median is indicated. The whiskers show the complete range. *P=0.0221 (Mann–Whitney *U-*test).

A striking difference in nucleoli architecture was observed between the NPM1 mutated lines and the OCI-AML2 control cells (Fig. 3A). In OCI-AML2 cells, imaging and line-scan analyses revealed that endogenous NPM1wt localised neatly to the nucleoli rim (Fig. 3B, presence of double peaks) and the nucleoli exhibit typical healthy circular morphology (Fig. 3C). In contrast, in OCI-AML3 and IMS-M2 cells, endogenous NPM1wt failed to localise to the nucleoli rim (Fig. 3B, presence of single peaks) and instead showed a diffuse distribution within the remaining nucleolar compartment. In addition, the nucleoli in these cells exhibited irregular and abnormal nucleolar morphology (Fig. 3C).

To further validate these findings, we imaged two additional NPM1wt AML cell lines, HL-60 and MOLM-13 (Fig. S2A–E). Consistent with what was seen for OCI-AML2 cells, endogenous NPM1 in these lines localised to the nucleoli rim and the nucleoli maintained a circular phenotype, unlike the diffuse NPM1 localisation and irregular nucleoli shape observed in the mutated cell lines. Quantitative analysis confirmed highly significant differences between NPM1wt and NPM1c+ cells in nucleolar rim intensity (*P*=0.0001) (Fig. 3B) and nucleolar circularity (*P*<0.0001) (Fig. 3C).

To extend these findings to a more clinically relevant context, we next examined six primary samples from individuals with AML. In NPM1 wild-type cells (*n*=3), NPM1 localised to the nucleoli rim and nucleoli appeared circular (Fig. 3Di). In contrast in NPM1c+ mutated samples (*n*=3), NPM1 was absent from the nucleoli rim, showing diffuse staining in the nucleolus, which appeared distorted (Fig. 3Dii). Quantification of images, using pixel density line scans, confirmed a highly significant difference in nucleolar rim intensity between NPM1wt and NPM1c+ cells (*P*≤0.0001) (Fig. 3E). To address whether the change in nucleoli morphology seen in NPM1 mutated cells influenced function, we assessed RNA polymerase I activity by measuring 45S rRNA transcription. Real-time RT-PCR analysis of 45S rRNA indicated that rRNA synthesis was significantly increased in NPM1 mutated cells (Fig. 3F).

### Reversible induction of aberrant nucleolar architecture – the impact of NPM1c+ expression in wild-type AML cells and NPM1wt expression in mutated AML cells

**Fig. 4.**
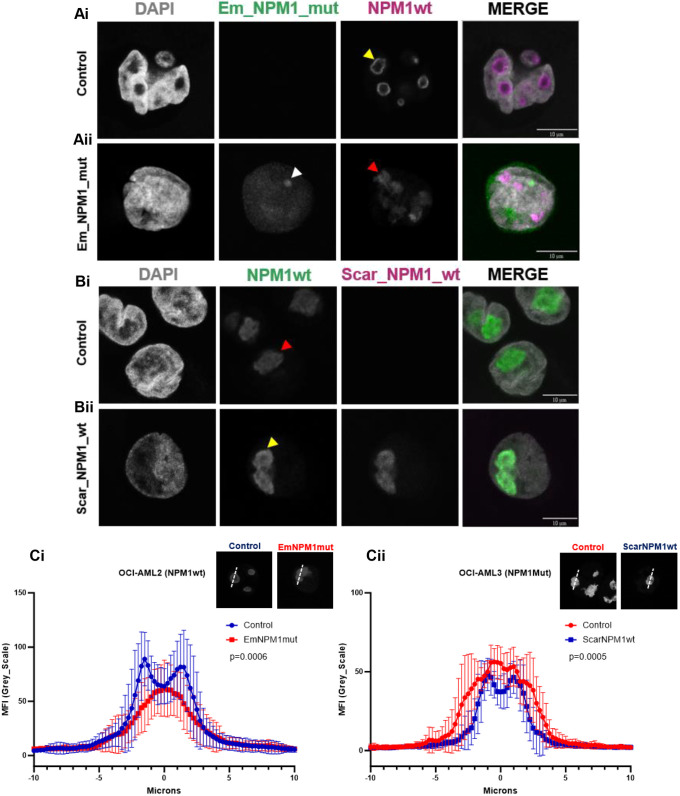
**Reversible induction of aberrant nucleolar architecture – the impact of NPM1c+ expression in wild-type AML cells and NPM1wt expression in mutated AML cells.** (A) OCI-AML2 and (B) OCI-AML3 cells were transfected with Em_NPM1_mut or Scar_NPM1_wt, respectively, for 24 h and stained with DAPI and NPM1wt antibody. Yellow arrowheads indicate NPM1wt nucleoli phenotype (Ai,Bii). Red arrowheads indicate NPM1 mutant nucleoli phenotype (Aii,Bi). The white arrowhead indicates NPM1 mutant protein aggregate (Aii). (C) NPM1wt localisation at the nucleolus of OCI-AML2 cells (Ci) and OCI-AML3 cells (Cii), %ΔMFI (High/Low; MFI, mean fluorescence intensity) *P*=0.0006 and *P*=0.0005 (unpaired two-tailed *t*-test). Mean of 20 nucleoli for each condition: bars, s.d.

To dissect the relationship between NPM1c+ expression and aberrant nucleolar architecture – particularly nucleolar rim integrity, we conducted reciprocal mutant and wt expression experiments exploiting the genetically distinct OCI-AML2 and OCI-AML3 cell lines. When NPM1c+ was expressed in the OCI-AML2 cells, it forced a shift from normal nucleolar architecture to an aberrant phenotype characterised by the loss of the nucleolar rim (Fig. 4A). Conversely, the introduction of NPM1wt into the OCI-AML3 cells, restored nucleolar architecture and reformed the nucleolar rim (Fig. 4B). The changes in NPM1 localisation, quantified via line scan analyses, were highly significant (*P*=0.006 and 0.005) (Fig. 4C).

These findings indicate that loss of nucleolar integrity in NPM1-driven AML does not precede mutation of NPM1, but rather, that NPM1 is the initial cause – but importantly is also reversible.

Additionally, during our investigation, we discovered an unexpected novel feature of NPM1c+ mutants – following transfection of the Em_NPM1_mut construct into the OCI-AML2 cells, we consistently observed that a portion of NPM1c+ assembled into protein aggregates (Fig. 4Aii, white arrowhead). These aggregates were not formed when Scar_NPM1_wt was introduced into OCI-AML3 cells.

### Mutant NPM1 protein forms distinct pools of protein aggregates

**Fig. 5.**
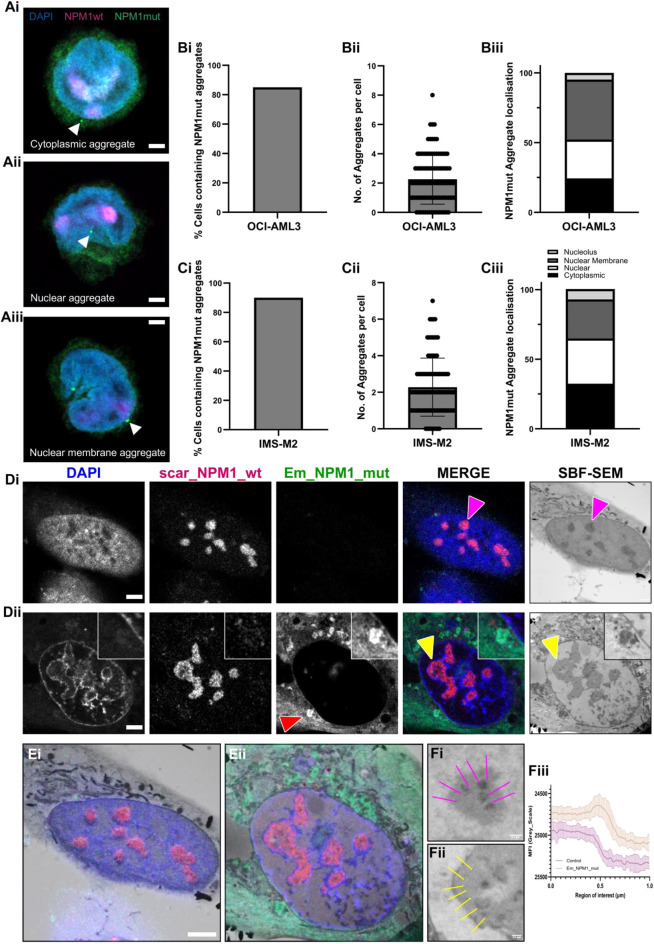
**NPM1 mutant protein forms distinct pools of protein aggregates.** (A) NPM1 mutated OCI-AML3 cells were stained with DAPI and antibodies against NPM1 mutant and NPM1wt. Images show representative examples of aggregate localisation (indicated by white arrowheads) in the cytoplasm, in the nucleus and at the nuclear membrane. Fluorescence image acquisition was performed using a ZEISS LSM 900 confocal microscope in Airyscan mode with a 100× oil immersion objective. (B,C) Aggregate quantification was performed by collating images from multiple slices along the *z*-axis and analysed as maximum intensity projections. Columns, mean data from 100 representative cells: bars, s.d. (D) mScarlet-tagged NPM1wt knock-in HeLa cells were subjected to 24 h Em_NPM1_mut transfection (Dii) or transfection control (Di). Light microscope images are shown in the first four panels with EM overlays in the far-right panels. The red arrowhead in Dii indicates an NPM1 mutant aggregate. The yellow arrowheads in Dii indicate an electron-dense region surrounding the nucleoli that is not seen in control cells (pink arrowheads). (E) 3D modelling of EM images using Amira software demonstrates clear registration with the original LM images. (F) Electron density measured from the nucleolus to nucleoplasm in control cells (Fi) or Em_NPM1_mut expressing cells (Fii) was quantified using the line scan tool in Fiji (Fiii), bars, s.d. Images representative of three experimental repeats. Scale bars: 1 µm (A); 5 µm (D,E).

To investigate whether the protein aggregates formed by expressing Em_NPM1_mut were inherent to NPM1 mutated cells, or merely an artifact of overexpression, we re-examined our extensive collection of fluorescence images. Strikingly, we consistently observed NPM1 mutant protein aggregates, in the NPM1 mutated cell lines but also, importantly, in the samples from individuals with AML (Fig. S3A–C). The aggregates appeared to represent a distinct pool of mutant NPM1 protein as they were not recognised by the NPM1wt-specific antibody. Furthermore, when we ectopically expressed the mutant NPM1 construct in HEK-293T and HeLa cells, we observed similar aggregate formation, which was absent when we ectopically expressed the NPM1wt construct in the same cell lines (Fig. S3D,E). For the first time, we report that the majority of cells, naturally expressing NPM1c+ mutations contain NPM1c+ aggregates (OCI-AML3 85%, and IMS-M2 90%), and that aggregates were seen in all cells of both of the primary samples analysed. The number of aggregates per cell was 2.28±1.58 and 2.26±1.7 in the two NPM1 mutated cell lines and 4.1±1.8 and 2.45±1.19 in both of the NPM1 mutated primary samples (mean±s.d.). Curiously, we noticed that aggregates were not restricted solely to one cellular compartment. A total of 24–32.5% of aggregates were in the cytoplasm and 28–32.5% located in the nucleus, for both mutant cells lines (Fig. 5A–C). Notably, a large fraction (28–43%) were located on the cytoplasmic interface of the nuclear membrane. Distribution of the aggregates in the NPM1 mutated primary samples differed slightly, with most of the aggregates (53–55%) localised to the nucleus (Fig. S3F–K). Analogous to what was seen in the cell lines, a large fraction of the aggregates appeared to be tethered to the cytoplasmic side of the nuclear membrane. The significance of this variability remains elusive but will be the subject of future study.

Given that most nucleolar components are dense enough to be visualised by electron microscopy (EM), we next explored whether these mutant NPM1 aggregates could be observed at the ultra-structural level. We used SuperCLEM (Booth et al., 2019), an elegant multimodal imaging technique allowing rare events (such as aggregates) to be captured using both super resolution light microscopy (LM) and EM.

**Fig. 6.**
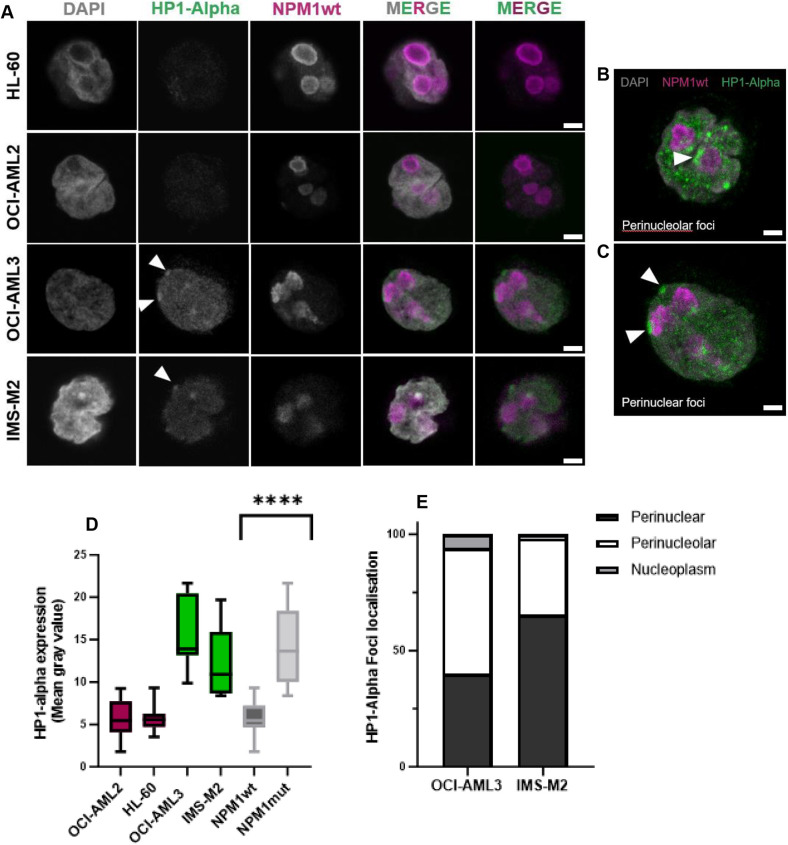
**Perinuclear and perinucleolar heterochromatin disorganisation in NPM1 mutated cells.** (A) AML cell lines were stained with DAPI and antibodies against HP1-α and NPM1wt. White arrowheads indicate HP1-α foci in NPM1 mutated OCI-AML3 and IMS-M2 cells. (B,C) White arrowheads indicate perinucleolar and perinuclear HP1-α foci localisation in OCI-AML3 cells. (D) Total HP1-α expression in the nucleus of AML cell lines was quantified using the threshold tool in Fiji. NPM1 wild-type cell lines in magenta, NPM1 mutant cell lines in green. Columns, mean of 10 nucleoli for each cell line: The box represents the 25–75th percentiles, and the median is indicated. The whiskers show the complete range. ****P=0.0001 (unpaired two-tailed *t*-test). (E) HP1-α quantification was performed by collating images from multiple slices along the *z*-axis and analysed as maximum intensity projections. Data from 10 representative cells. Scale bars 2.5 µm (A); 1 µm (B,C).

We identified and examined cells of interest – those expressing only NPM1wt as controls (Fig. 5Di) and those expressing Em_NPM1_mut (Fig. 5Dii). Aggregates were clearly visible in the cells expressing mutant NPM1 (red arrowhead). We prepared these for SuperCLEM and revisited our chosen cells using serial block face scanning electron microscopy (SBF-SEM) (Fig. 5D, right panels). Clear registration could be achieved between LM and EM images, confirming re-location of not only the same cells, but same area of each cell (Fig. 5E). SuperCLEM revealed several structural differences between control and mutant NPM1 expressing cells. First, registration of optical (light) and physical (EM) sections allowed us to visualise areas of the cell corresponding to aggregates (Fig. 5Dii, red arrowhead). Clear, electron-dense structures were present at sites of registered aggregate sites (Fig. 5Dii, magnified inset). Second, there was a clear shift in the electron density of the nucleoplasm, between control and NPM1 mutant samples. Third, linked to the second point, in the Em_NPM1_mut-expressing cells, we observed the presence of a density surrounding the nucleolus (Fig. 5Dii, far right panels, yellow arrowheads) typically reminiscent of heterochromatin staining, which was not seen in the control cells (Fig. 5Di, far right panels, pink arrowheads). This was quantifiable using a line scan analysis measuring the pixel densities between the nucleolar body, through the rim and into the nucleoplasm (Fig. 5F). The presence of the ‘shoulder’ in Fig. 5Fiii reflects the electron-dense region. To more directly validate this initial observation, we next sought to determine whether the presence of an NPM1 mutation causes heterochromatin rearrangement, using HP1-α (also known as CBX5) as a marker. We stained and imaged NPM1 wild-type OCI-AML2 and HL-60 cells and NPM1 mutated OCI-AML3 and IMS-M2 cells, revealing a far more intense staining in the mutant cell lines (Fig. 6A,D), predominantly in the form of characteristic foci (Fig. 6B,C) that localised almost explicitly to perinuclear or perinucleolar regions (Fig. 6E), consistent with heterochromatin.

## DISCUSSION

Mutations in exon 12 of the *NPM1* gene were first identified almost 20 years ago and represent one of the most common genetic alterations in AML (Falini et al., 2005). The mutations are always heterozygous, suggestive of an essential role for wild-type NPM1, although preferential transcription of the mutant allele has been reported (Bailey et al., 2020). The NPM1c+ mutation has important clinical and prognostic implications, and the focus of much research has centred on the mis-localisation of the mutated protein from the nucleolus to the cytoplasm (Falini et al., 2020; Papaemmanuil et al., 2016). Here, we report that NPM1wt subcellular localisation is also greatly impacted in NPM1 mutated cells. Specifically, NPM1 dissociates from its usual location at the nucleoli rim and instead exhibits a diffuse staining pattern in the nucleoli with resultant breakdown of nucleoli structure. We also found that rRNA synthesis was significantly increased in NPM1 mutated cells, offering a causal link between morphology and function. Other groups have reported an upregulation of rRNA processing and ribosomal biogenesis in NPM1 mutated AML via the ribosomal protein S2 (RPS2), suggesting a possible target for therapeutic intervention (Feng et al., 2023). We also report that the aberrant nucleoli phenotype in NPM1 mutated cells is reversible and, conversely, that aberrant nucleoli phenotype can be induced in NPM1wt cells. To our knowledge this is the first demonstration of this kind of nucleoli architecture manipulation in AML cells but is of significance as we search for new strategies to treat NPM1-driven AML, including the potential of nucleoli as vulnerable targets. Morphologically, nucleoli consist of three distinguishable phase separated regions, the fibrillar centre, the dense fibrillar component and the granular component. More recently, a fourth nucleoli sub-compartment, the nucleoli rim, has been identified. We demonstrate that under normal physiological conditions NPM1 locates to the nucleoli rim, but in NPM1 mutated cells this rim localisation is lost, resulting in distorted nucleoli. The localisation of NPM1 to the nucleoli rim is seemingly dependent on the amount of NPM1wt in the nucleolus, as we report that both knockdown of NPM1wt in NPM1wt cells and mis-localisation of a portion of NPM1wt to the cytoplasm in NPM1c+ cells result in nucleoli distortion. Studies have implicated NPM1 in maintaining a normal nucleolar structure through its interaction with ribosomal subunit precursors, and cells depleted of NPM1, exhibit deformed nucleoli and rearrangement of peri-nucleolar heterochromatin. Indeed, our ultrastructural analysis, using SuperCLEM, supports this, as the expression of NPM1c+ mutant caused apparent changes in chromatin organisation as observable by both super resolution light microscopy (DAPI staining) and also at the underlying EM level, as an electron-dense region surrounding the nucleolus, typically reminiscent of heterochromatin staining. This was also supported by HP1-α antibody staining, a commonly used heterochromatin marker. Disruption of the nucleolus and subsequent dysregulated ribosome biogenesis has been linked to tumour initiation and cancer progression, implicating treatments that affect nucleoli as a potential therapeutic approach (Orsolic et al., 2016). In fact, it has been suggested that the nucleoli of NPM1 mutated AML cells might be particularly susceptible to drugs that induce a nucleolar stress response due to the partial depletion of NPM1 caused by both haploinsufficiency and retention of a fraction of NPM1wt by NPM1c+ in the cytoplasm (Falini et al., 2015). The nucleolar stress-inducing drug Dactinomycin has also been shown to preferentially target NPM1 mutated cells (Gionfriddo et al., 2021). We have previously reported that etoposide- or cytarabine-induced DNA damage alters the subcellular localisation of mutated NPM1 back to a predominantly nucleolar distribution (Bailey et al., 2019). Thus, the induction of nucleolar stress has emerged as a potential therapeutic strategy for NPM1 mutated AML.

The N-terminus of NPM1 contains a self-oligomerisation core that is responsible for the formation of NPM1 pentamers, and the oligomerisation state of NPM1 is crucial for its normal biological functions (Mitrea et al., 2018; Okuwaki et al., 2023). It is therefore important to explore the molecular links between oligomerisation and NPM1-driven leukaemia. Our high-resolution imaging consistently revealed the presence of distinct pools of NPM1 mutant protein aggregates in both NPM1 mutated cell lines and primary samples. Aggregates were also present when we ectopically expressed mutant NPM1 in HEK-293T and HeLa cells, but not with NPM1wt ectopic expression, suggesting that the mutated C-terminal of NPM1c+ is responsible for aggregate formation. The significance of the variability in the localisation of the aggregates remains unclear but is likely related to how and when they are assembled and warrants further study.

Our SuperCLEM analysis revealed that the aggregates co-register to electron-dense areas. These could correspond to self-assembling phase-separated compartments or could be a result of abnormal nucleolar disassembly. EM also revealed a redistribution of chromatin and recruitment of an electron-dense nucleoli surface, potentially indicative of a change in chromatin from a more open (euchromatic) to a more closed (heterochromatic) state. This finding was supported through complementary fluorescence imaging experiments, scoring for HP1-α abundance and distribution in the presence and absence of NPM1c+, revealing a prominent increase in both perinuclear and peri-nucleolar HP1-α foci, but only in NPM1 mutant-expressing cells. Interestingly, by means of complementary biophysical techniques, studies have linked the aggregation propensity of distinct regions of the improperly folded C-terminal domain to leukaemogenesis in NPM1-mutated AML; by comparing the conformational and aggregate-forming ability of the entire C-terminal domains of NPM1wt and NPM1c+ the authors reported that only the C-terminus on NPM1c+ and not NPM1wt can form amyloid-like aggregate assemblies (Di Natale et al., 2020; Scognamiglio et al., 2016).

Here, for the first time, we report NPM1 mutant protein aggregate formation and a distinct nucleoli phenotype in NPM1 mutated AML cells, both of which represent potential therapeutic vulnerabilities. The fact that this particular pool of mutant NPM1 cannot readily form multimers with NPM1wt is of crucial importance, therefore the spatiotemporal origins (and disassembly) and biochemical profiles of these aggregates need to be carefully studied in future studies.

## MATERIALS AND METHODS

### Cells

OCI-AML3, OCI-AML2, HL-60 and MOLM-13 cells were obtained from the Leibniz Institute (DSMZ, Braunschweig, Germany). IMS-M2 cells were a kind gift from Carolien Woolthuis, University of Groningen, The Netherlands. Cells were maintained in RPMI 1640 medium with 10% fetal calf serum (Thermo Fisher Scientific, Hampton, New Hampshire, USA) and 2 mM L-glutamine (Sigma-Aldrich, St Louis, Missouri, USA). HeLa and HEK-293T cells were obtained from DSMZ and maintained in DMEM (Thermo Fisher Scientific) with 10% FCS. Cultures were sustained at 37°C in 5% CO_2_, and all experiments were performed with cell lines in log phase. Monthly mycoplasma testing was performed using a PlasmoTest mycoplasma contamination detection kit (InvivoGen, Toulouse, France). Samples from individuals with AML were obtained with informed consent in accordance with the principles expressed in the Declaration of Helsinki and approval by the authors' institutional review board (REC# 06/Q2403/16). Informed consent was obtained from all subjects. Mononuclear cells were obtained by standard density gradient centrifugation from bone marrow or peripheral blood samples and cells were cryopreserved until use. The presence of NPM1 mutations were identified as previously described (Noguera et al., 2005).

### Constructs

Gene inserts were synthesised by GeneArt (Regensberg, Germany) as follows. For the NPM1wt insert, unique SacI (5′) and EcoRI (3′) sites were added to full length hsNPM1wt (NM_002520.7). For the NPM1 mutant insert, the C-terminal of the NPM1wt insert was replaced with the mutated sequence (5′-GATCTCTGTCTGGCAGTGGAGGAAGTCTCTTTAAGAAAATAG-3′). NPM1wt and NPM1 mutant inserts were cloned into the SacI and EcoRI sites of pmScarlet_C1 (Addgene #85042) and mEmerald-C1 (Addgene #53975) plasmids, respectively.

### Transfections

HeLa or HEK-293T cells in exponential growth were seeded in 12-well plates on glass coverslips and cultured overnight. Transfections were performed using Polyplus jetPRIME (VWR, Lutterworth, Leicestershire, UK) with either 0.8 µg of Scar_NPM1_wt or Em_NPM1_mut constructs. NPM1 wild-type OCI-AML2 and NPM1 mutant OCI-AML3 cells were nucleofected with 1 µg Em_NPM1_mut or Scar_NPM1_wt constructs respectively using Nucleofector Kit-T (cat# VCA-1002, Lonza, Basel, Switzerland) with program X-001 using a Nucleofector-2b device (Lonza). Construct expression and ectopic protein localisation was confirmed using fluorescence microscopy.

### Generation of fluorescently tagged NPM1 HeLa cells using CRISPR/Cas9-mediated gene editing

DNA oligonucleotides (forward, 5′-ACATGGACATGAGCCCCCTG-3′; reverse 5′-GATAGTTCTGGGGCCTCAGG-3′) used for gRNA synthesis were designed using the Benchling CRISPR gRNA Design Tool available at https://www.benchling.com/ (accessed on 15 May 2022) and ordered through Sigma. gRNA was cloned into the Bbs1 restriction site of the pX330-U6-Chimeric_BB-CBh-hSpCAas9-hGem (1/110) vector (Addgene #71707) before transformation into NEB^®^ 5-alpha Competent *E. coli* (New England Biolabs, Ipswich, MA, USA). DNA was prepared from single colonies before sequence verification using an Applied Biosystems™ (Waltham, Massachusetts, USA) 3130*xl* genetic analyser. Homology repair plasmids (HDR) designed to introduce mScarlet or mEmerald to the N-terminus of NPM1 using a pUC18 backbone were ordered from GenScript (Piscataway, New Jersey, USA). HeLa cells were nucleofected with the HDR plasmid and the gRNA cas9 plasmid and allowed to recover for 3 days before selection with 800 µg/ml G418 (Thermo Fisher Scientific) for 7 days. To remove antibiotic selection via the loxP sites, and allow transcription of tagged protein, cells were transfected with pMSCVpuro-Cre plasmid (Addgene #34564). Cells were then treated with 3 µg/ml puromycin (Thermo Fisher Scientific) for 3 days and single cell sorted into 96-well plates. Clones were screened by PCR genotyping.

### RNA interference

For RNAi treatments, HeLa or HEK-293T cells in exponential growth were seeded in 12-well plates on glass coverslips and cultured overnight. Transfections were performed using Polyplus jetPRIME with 15 nM siRNAs targeting both the wild-type and mutant NPM1 (5′-AAAGGTGGTTCTCTTCCCAAA-3′) (Hs_NPM1_7, SI02654960, Qiagen, Hilden, Germany) or AllStars negative control siRNA (cat. #1027281, Qiagen). Following siRNA-mediated interference, NPM1 knockdown was confirmed by western blotting and indirect immunofluorescence. For the rescue experiments, HEK-293T cells at 50% confluence were transfected with 15 nM NPM1 targeting siRNA (Hs_NPM1_7) or AllStars negative control siRNA. Following 2 days of siRNA-mediated interference, cells were transfected with 0.8 µg of Scar_NPM1_wt construct and cultured for 24 h before validation by indirect immunofluorescence.

### Indirect immunofluorescence and microscopy

Primary antibodies against the indicated proteins were used as follows: NPM1wt, which reacts with the C-terminus of NPM1 (mouse monoclonal, #32-5200; Thermo Fisher Scientific), 1:60; NPM1 mutant which reacts with the C-terminus of mutant NPM1 (rabbit polyclonal, Thermo Fisher Scientific, #PA1-46356), 1:60; nucleolin (rabbit polyclonal, Abcam, Cambridge, UK, #ab22758), 1:100; anti-HP1-α (rabbit monoclonal, Abcam #109028), 1:250. Fluorescence-labelled secondary antibodies were applied at 1:400 (Jackson ImmunoResearch, Ely, UK). For immunofluorescence, cells were fixed in 3.5% paraformaldehyde for 15 min, permeabilised in 0.3% Triton X-100 for 5 min and blocked in 2% blocking solution (Thermo Fisher Scientific). Cells were incubated overnight with the primary antibodies washed in PBS and secondary antibodies were applied for 1 h before counter-staining with DAPI. Fluorescent image acquisition was performed using a Leica (Wetzlar, Germany) TCS SPE confocal microscope with a 63× oil immersion objective. Images were exported as TIFF files and imported into Fiji software (Schindelin et al., 2012) for final presentation.

### Confocal live cell microscopy

HeLa or HEK-293T cells were seeded in a glass bottomed cellview cell culture dish (Greiner, Kremsmunster, Austria) and transfected as described above with 0.8 µg Scar_NPM1_wt construct. Cells were counterstained in 1 µM SiR-DNA (Spirochrome, Thurgau, Switzerland) and imaged in Fluorobrite DMEM without Phenol Red (Thermo Fisher Scientific). Live-cell imaging was performed at 37°C and 5% CO_2_ using the Zeiss Cell discoverer 7 imaging system (Zeiss, Oberkochen, Germany). Optical settings were an objective lens magnification of 50× and an Optovar magnification of 0.5×. Image videos were generated using Fiji software.

### Image analysis

NPM1 localisation in the nucleolus was quantified using the line scan tool in Fiji software. Nucleoli circularity was determined using the threshold tool. Aggregate and HP1-α quantification was performed by collating images from multiple slices along the *z*-axis with a sampling depth of 2 µm and presented as maximum intensity projections. For EM line scan, 5–10 1 µm line scans were used per nucleoli (4–8 nucleoli in total). Pixel densities were retrieved with the line scan origin located in the nucleolus and terminating in the nucleoplasm, with the nucleolar-to-nucleoplasm interface being approximately mid-line scan (0.5 µm). Pixel densities were recorded every 10 nm.

### Western blot analysis

HeLa cells were subjected to 3 or 6 days NPM1 siRNA interference. Cell lysates were prepared, separated by SDS-PAGE, and transferred to nitrocellulose membranes. Primary detection antibodies were mouse anti-NPM1 (1:1000; Thermo Fisher Scientific, #32-5200), rabbit anti-nucleolin (1:1000; ab22758), rabbit monoclonal anti-actin (1:5000; ab179467), and mouse monoclonal anti-actin (1:100; ab8226) (all from Abcam). Secondary detection antibodies were donkey anti-mouse-IgG conjugated to IRDye 680 (#926-68072) and donkey anti-rabbit-IgG conjugated to IRDye 680 (#926-68073) from LI-COR (Lincoln, Nebraska, USA). Band quantification was determined using Fiji. See Fig. S4 for unprocessed images of blots.

### SuperCLEM

mScarlet-tagged NPM1wt knock-in HeLa cells were seeded onto glass-bottomed, gridded dishes (MatTek Corporation, Ashland, Massachusetts, USA) and transfected with Em_NPM1_mut construct or transfection control. Following a 24 h expression period, cells of interest were identified using a Zeiss LSM900 confocal microscope mounted with Airyscan module. Cells were located and their position mapped using transmitted light to visualise reference coordinates. The SuperCLEM processing method was an adapted version of a previously established protocol (Booth et al., 2016). Images were acquired using a Gatan 3View serial block face system (Gatan, Pleasanton, California, USA) installed on a FEI Quanta 250 FEG scanning electron microscope (FEI Company, Hillsboro, Oregon, USA). Images were collected at a magnification of 5.7 k, voxel size of 12×12×60 nm and chamber pressure of 70 Pa at 4 kV. Post acquisition analysis was performed using Amira software (Thermo Fisher Scientific).

### RNA extraction, reverse transcription and quantitative RT-PCR

Extraction of RNA from AML cell lines and primary samples from human individuals was achieved using a QIAamp RNA Blood Mini kit (Qiagen, Hilden, Germany) according to the manufacturer's instructions. cDNA was synthesised from 2 µg total RNA using MML-V Reverse Transcriptase (Invitrogen, Waltham, MA, USA), with a reaction mixture of 5X First Strand Buffer (Invitrogen), 25 mM dNTPs (Invitrogen), 0.1 M DTT (Invitrogen) and random primers 3 µg/µl (Invitrogen). Transcript copy numbers were measured using a 7500 Fast Real-Time PCR analyser (Applied Biosystems). Reaction mixtures were assembled using 5 ng of cDNA, SYBR Green Fast PCR Master Mix (Thermo Fisher Scientific) and 1 µM forward and reverse primers (Integrated DNA technologies, Coralville, Iowa, USA). Samples were tested in triplicate and β2-microglobulin was used as a reference gene for data normalisation. Primer sequences were as follows: B2 M Forward (5′-GAGTATGCCTGCCGTGTG-3′); B2 M Reverse (5′-AATCCAAATCGCGCATCT-3′); 45S Forward (5′-CCTGCTGTTCTCTCGCGCGTCCGA-3′); 45S Reverse (5′-AACGCCTGACACGCACGGCACGGAG-3′). 45S gene expression was calculated using the 2^(−ΔCT) method.

### Calculations and Statistics

Unpaired two-tailed *t*-tests were performed using GraphPad Prism version 10.0.2 for Windows, GraphPad Software, Boston, MA, USA. *P*≤0.05 were considered to represent significance.

## Supplementary Material

10.1242/joces.263553_sup1 Supplementary information
