# Supplementary material for: Abnormal nucleoli architecture and aggregate formation in nucleophosmin mutated acute myeloid leukaemia
Source: J Cell Sci. 2025 May 21;138(10):jcs263553. doi: 10.1242/jcs.263553 (PMC12148033; doi:10.1242/jcs.263553)
Supplement: Supplementary information [file joces-138-263553-s1.pdf]

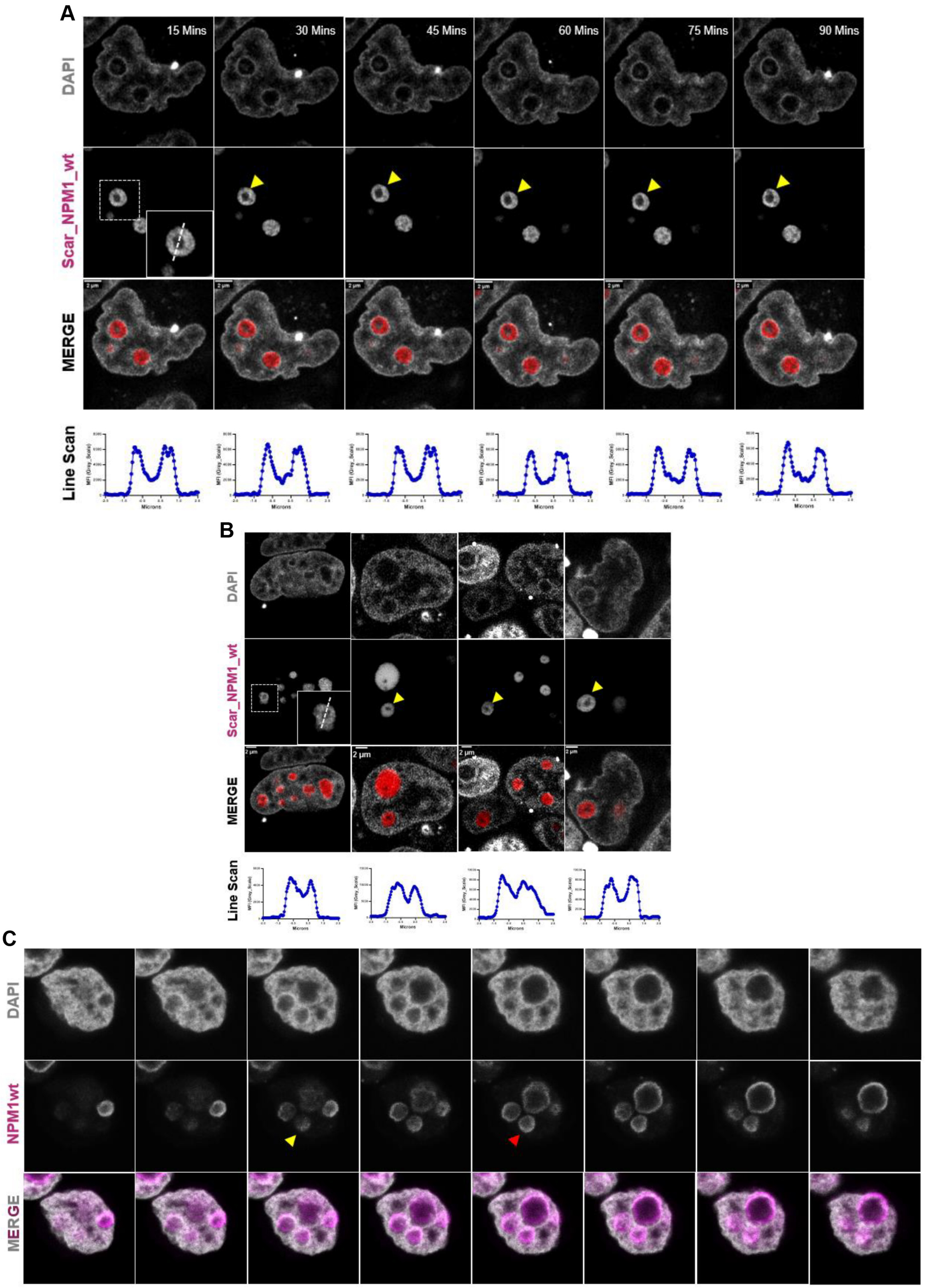

Journal of Cell Science • Supplementary information

Fig. S1. NPM1 wild-type nucleoli rim localisation in living cells.(A) HeLa or **(B)** HEK**-**293T cells were transfected with Scar_NPM1_wt construct and counterstained with SiR-DNA before live imaging. Images shown in (A) are stills from the same cell imaged every 15 mins over a period of 90 mins. Images shown in (B) are different cells imaged at a single timepoint. **(C)** NPM1 wild-type HL-60 cells were stained with DAPI and an antibody against NPM1wt. Images shown are multiple slices along the z-axis.

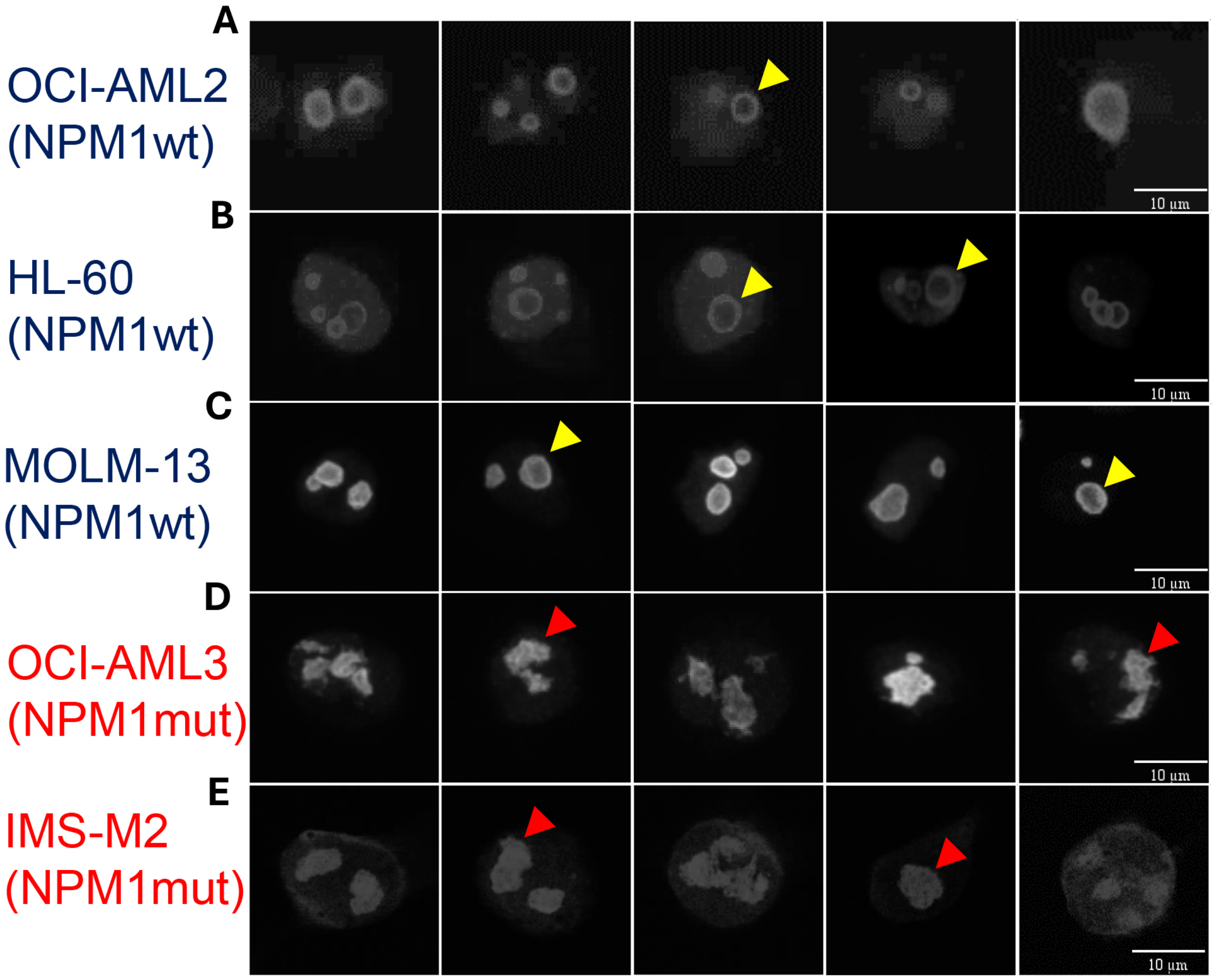

Fig. S2. NPM1 mutated cells lack a nucleoli rim and have distorted nucleoli compared to NPM1 wild-type cells.

AML cell lines were stained using an antibody against NPM1wt. Yellow arrows indicate NPM1wt localisation to the nucleoli rim in 3 NPM1 wild-type cell lines **(A, B and C)**. Red arrows indicate a lack of nucleoli rim and distorted nucleoli in NPM1 mutated OCI-AML3 and IMS-M2 cell lines **(D and E)**. The two far right figures in **(D)** are reproduced from Fig. 3C in the main paper.

Journal of Cell Science • Supplementary information

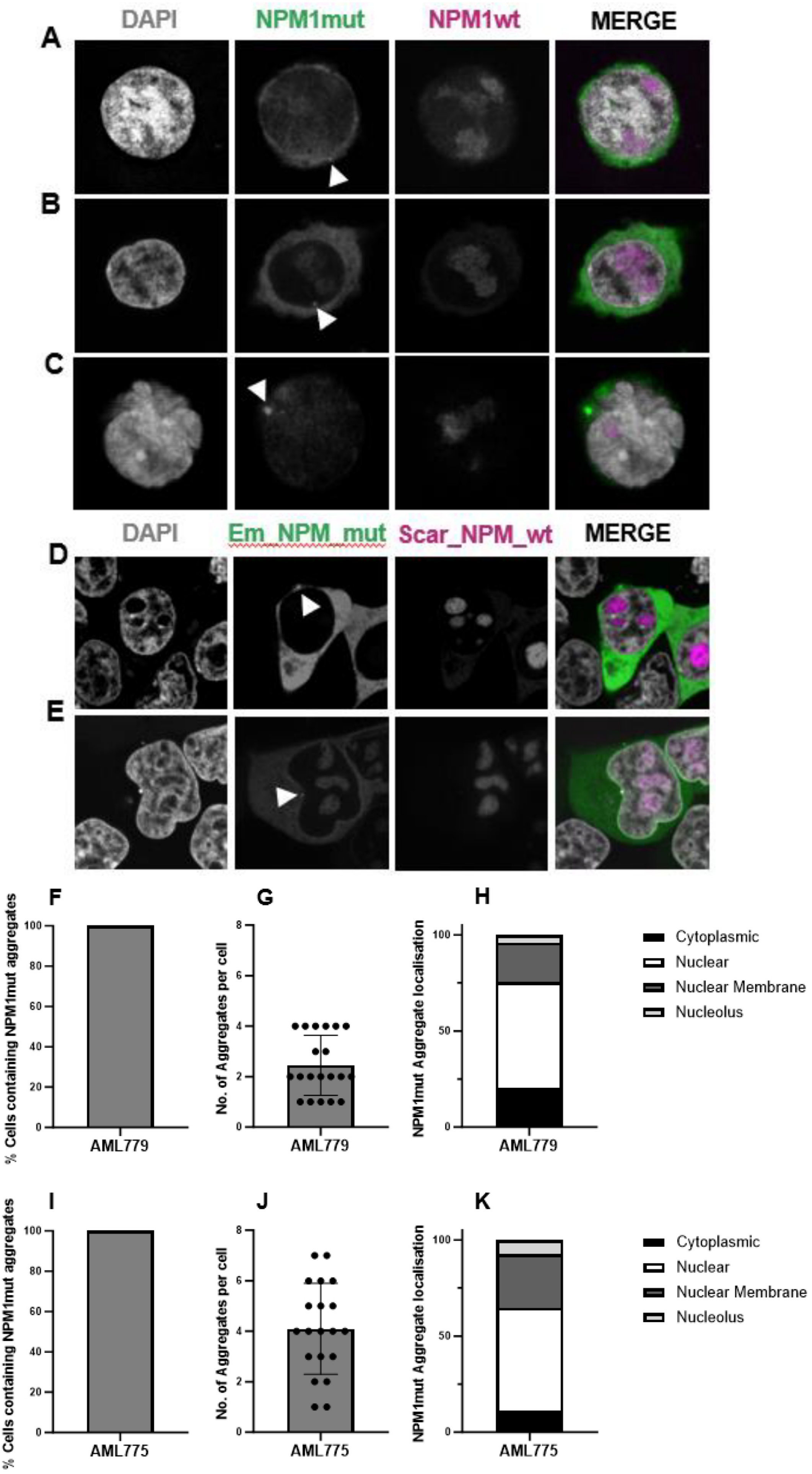
DAPI
NPM1mut
NPM1wt
MERGE
A
B
C
DAPI
Em_NPM_mut
Scar_NPM_wt
MERGE
D
E
F
% Cells containing NPM1mut aggregates
AML779
G
No. of Aggregates per cell
AML779
H
NPM1mut Aggregate localisation
AML779
Cytoplasmic
Nuclear
Nuclear Membrane
Nucleolus
I
% Cells containing NPM1mut aggregates
AML775
J
No. of Aggregates per cell
AML775
K
NPM1mut Aggregate localisation
AML775
Cytoplasmic
Nuclear
Nuclear Membrane
Nucleolus

Fig. S3. NPM1 mutant protein forms distinct pools of protein aggregates.

(A) OCI-AML3 **(B)** IMS-M2 or **(C)** primary cells were stained with DAPI and antibodies against NPM1 mutant and NPM1wt. Figures reproduced from Fig. 3A and Fig. 3D in the main paper. **(D)** HEK-293T and **(E)** HeLa cells were transfected with Scar_NPM1_wt and Em_NPM1_mut constructs for 24 hrs. White arrows indicate NPM1 mutant protein aggregates. Figures reproduced from Fig. 2A and Fig. 2B in the main paper. **(F), (G), (H), (I), (J) and (K)** NPM1 mutated primary samples were stained with DAPI and antibodies against NPM1 mutant and NPM1wt. Aggregate quantification was performed by collating images from multiple slices along the z-axis and analysed as maximum intensity projections. Columns, mean data from 20 representative cells: bars, s.d.

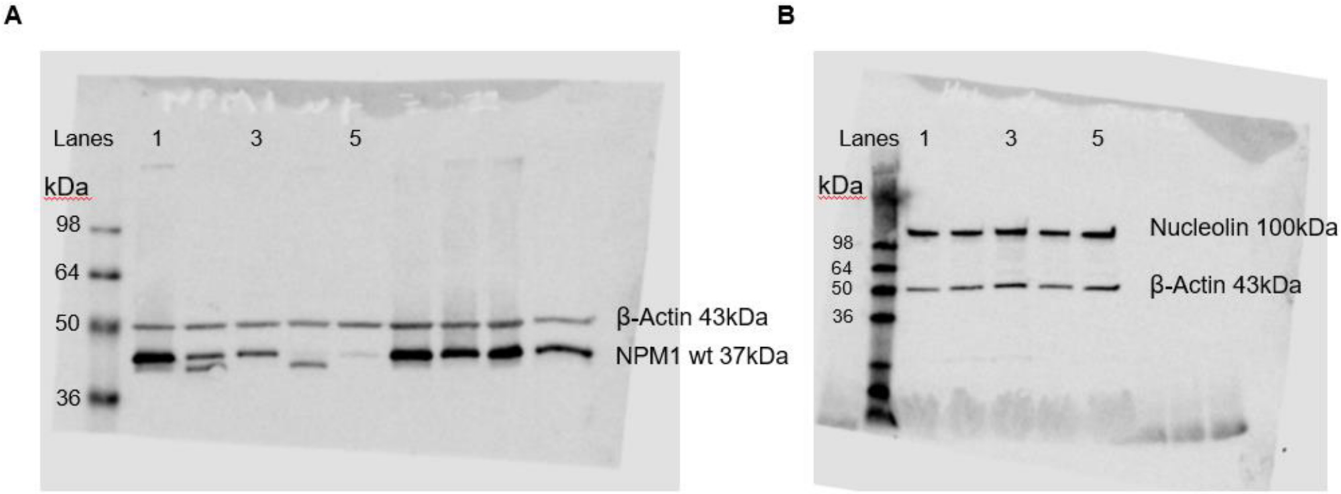

Fig. S4. siRNA targeted NPM1 depletion – Western blot transparency. **(A)** NPM1wt and **(B)** nucleolin protein expression in Hela cells following 3 or 6 days NPM1wt siRNA interference. Lanes 1 (Control siRNA), lanes 3 (3 days NPM1wt siRNA) and lanes 5 (6 days NPM1wt siRNA) were used in Fig. 1A.

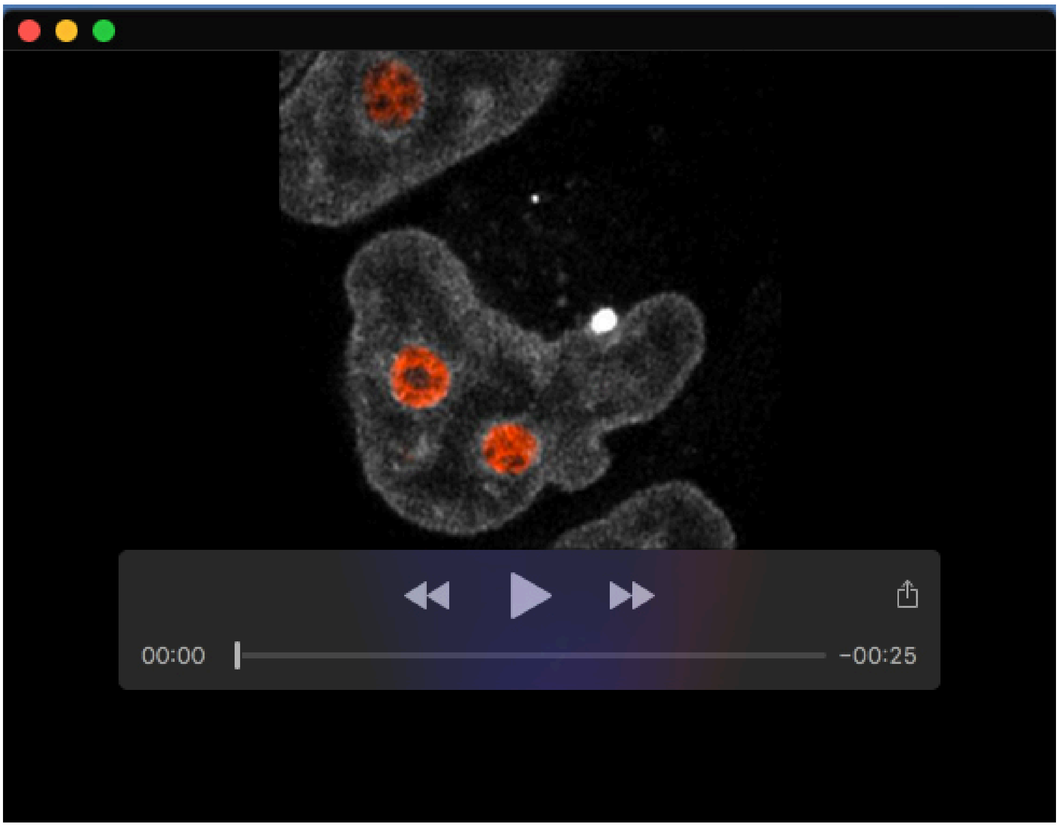

Movie 1. NPM1 wild-type nucleoli rim localisation in living cells. HeLa **cells** were transfected with Scar_NPM1_wt (Red) construct and counterstained with SiR-DNA (Grey) before live imaging. Movie shown is of the same cell imaged every 15 mins over a period 6 hrs.

Journal of Cell Science • Supplementary information
